# Lead Us Not into Tanktation: A Simulation Modelling Approach to Gain Insights into Incentives for Sporting Teams to Tank

**DOI:** 10.1371/journal.pone.0080798

**Published:** 2013-11-29

**Authors:** Geoffrey N. Tuck, Athol R. Whitten

**Affiliations:** 1 CSIRO Computational Informatics, CSIRO Marine and Atmospheric Research and the CSIRO Digital Productivity and Services Flagship, Hobart, Tasmania, Australia; 2 Mezo Research, Melbourne, Victoria, Australia; National Research & Technology Council, Argentina

## Abstract

Annual draft systems are the principal method used by teams in major sporting leagues to recruit amateur players. These draft systems frequently take one of three forms: a lottery style draft, a weighted draft, or a reverse-order draft. Reverse-order drafts can create incentives for teams to deliberately under-perform, or tank, due to the perceived gain from obtaining quality players at higher draft picks. This paper uses a dynamic simulation model that captures the key components of a win-maximising sporting league, including the amateur player draft, draft choice error, player productivity, and between-team competition, to explore how competitive balance and incentives to under-perform vary according to league characteristics. We find reverse-order drafts can lead to some teams cycling between success and failure and to other teams being stuck in mid-ranking positions for extended periods of time. We also find that an incentive for teams to tank exists, but that this incentive decreases (i) as uncertainty in the ability to determine quality players in the draft increases, (ii) as the number of teams in the league reduces, (iii) as team size decreases, and (iv) as the number of teams adopting a tanking strategy increases. Simulation models can be used to explore complex stochastic dynamic systems such as sports leagues, where managers face difficult decisions regarding the structure of their league and the desire to maintain competitive balance.

## Introduction

Major sporting bodies such as the National Football League (NFL), National Basketball Association (NBA), Major League Baseball (MLB) and the Australian Football League (AFL) have adopted player recruiting systems whereby the best amateur players in a particular year are chosen by clubs in reverse order to their finishing position in that year [1]–[4]. The reverse-order draft system has the benefit of allowing clubs with the poorest win-loss record in a season access to the most highly-rated amateur players. Consequently, low-ranking clubs gain the best opportunity to improve their player lists, which is then expected to increase the likelihood of on-field success. The inverse is also true: the assignment of lower draft picks to successful clubs is expected to weaken their player lists and reduce their chances of success. The desired effect of this system is to improve competitive balance, i.e. the long-term equalisation of the participating clubs, as far as both success and failure are concerned [1], [5]–[7]. Competitive balance has been viewed as a desirable management objective of sporting leagues as it enables poor performing clubs the opportunity to improve both on and off-field, and prevents high performing and richer clubs from dominating over long periods [8]. Reverse-order draft systems can be ineffective though, in terms of competitive balance, if restraints on player transfers are not in place following drafting [7], [9]–[11].

Controversially, reverse-order draft systems can create incentives to lose games [12]–[16]. As a sporting season nears completion, teams that are highly unlikely to participate in the finals (known as playoffs in some leagues), have an incentive to under-perform in order to obtain higher draft picks and therefore better players (to shirk, or to tank; from here on referred to as tanking). While deliberate under-performance is generally denied by sporting clubs and league management bodies, there is the legitimate potential for clubs to adopt strategies that will weaken their performance and reduce their chances of winning games. Such strategies might include playing young and inexperienced players in preference to older players, or resting injured players in preparation for the following season. These strategies are unlikely to be adopted by clubs that have some prospect of winning the championship or premiership (from here on referred to as premiership) in a given year. The result is a reduction in the team’s productivity or strength, a lower standing compared to other teams, and an increase in the potential to obtain higher draft picks than the team would have obtained otherwise [17].

For the NBA, Taylor and Trogdon [12] show an increased likelihood of non-playoff teams to lose under the reverse-order draft system. When all non-playoff teams had an equal chance of receiving the best player through a draft lottery system, this increase in under-performance was no longer found. Analyses by Borland, Chicu, and Macdonald [15] indicate there is little evidence for such deliberate under-performance in the AFL. The authors argue the incentive to deliberately under-perform is lower in the AFL than in other sporting leagues. They suggest it is more difficult to determine high quality players in the AFL, and that because fewer players are utilised on the field (or court) in the NBA (5 compared to 18), the influence of these players on team success is much greater. Despite this, speculation of deliberate under-performance in the AFL is commonplace, particularly in the final games of each season [15].

Reverse-order draft systems may also lead to habitually mid-ranked teams becoming ‘stuck’. Over the mid- to long-term, teams that do not ‘bottom out’, and receive high draft picks, may experience less success compared to teams that cycle between the bottom and top of the premiership rankings. Lenten [18] provides a statistical analysis of the cycles of AFL teams and shows that some teams cycle while others do not. This cycle of increasing and decreasing success, and stability of mid-ranked teams, has not previously been explored through a simulation model of sporting leagues.

El Hodiri and Quirk [19], in their foundational work on modelling major sporting leagues, recognise the dynamic nature of sporting competition and focus on steady-state solutions of the impacts of economic factors, such as gate-revenue, drawing potential and player trading, and on the distribution of player strength amongst teams and measures of competitive balance. Their model assumes a fixed total amount of playing skill every year which is distributed deterministically to clubs according to a reverse-order draft system, and skills depreciate linearly once players have been recruited. Quirk and El Hodiri [20] extend the model to allow variation in player ability over time, but find that many of the steady-state results no longer hold. Other model refinements have followed, but they too focus on equilibria and steady-state solutions [21]–[24].

Tuck and Whitten [25] introduced a stochastic non-equilibrium simulation model of a win-maximising sporting league: here referred to as Sports Synthesis. Sports Synthesis was used to explore how common amateur drafting systems of major sporting leagues influence incentives to tank. The model explicitly considers the dynamics of amateur player draft systems by including error in player draft selection, variable initial player ability, non-linear time-varying productivity of players as they progress through their career, and deliberate under-performance of clubs. The modelling framework shows how alternative draft systems can be compared against common performance measures and allow the trade-offs in performance to be explicitly considered by sports managers. In addition, the model is used to illustrate how new player draft systems can be developed, analysed, and explored for weaknesses that could be exploited by clubs.

In this paper we explore the behaviour of the model of Tuck and Whitten [25] which has been parameterised to be similar to the AFL. The model’s dynamic equations are iterated over a number of years and summary statistics are provided. We do not attempt to find analytic solutions assuming a steady-state. In this respect, the model is similar to the decision theory models used in biological resource assessment, such as those used to estimate the status of fish stocks and to evaluate alternative resource management strategies [26], [27]. In these fields, non-equilibrium methods are more commonly used to assess resource status and impacts of management decisions than in sport [28], [29]. We show how particular league characteristics can influence the dynamics of team success. In addition, we consider how characteristics, such as the number of teams in the league and draft uncertainty, can induce incentives for teams to deliberately under-perform.

## Methods

In this section the dynamic stochastic simulation model of the amateur player drafting process originally proposed by Tuck and Whitten [25] is described in more detail. A key assumption of the model structure is that a player’s productivity can be specified as a function of the player’s draft number and their age (or alternatively, the number of seasons played). The player productivity function defines the player’s ability relative to those of other players of different draft numbers and different ages [30].

**Figure 1:**
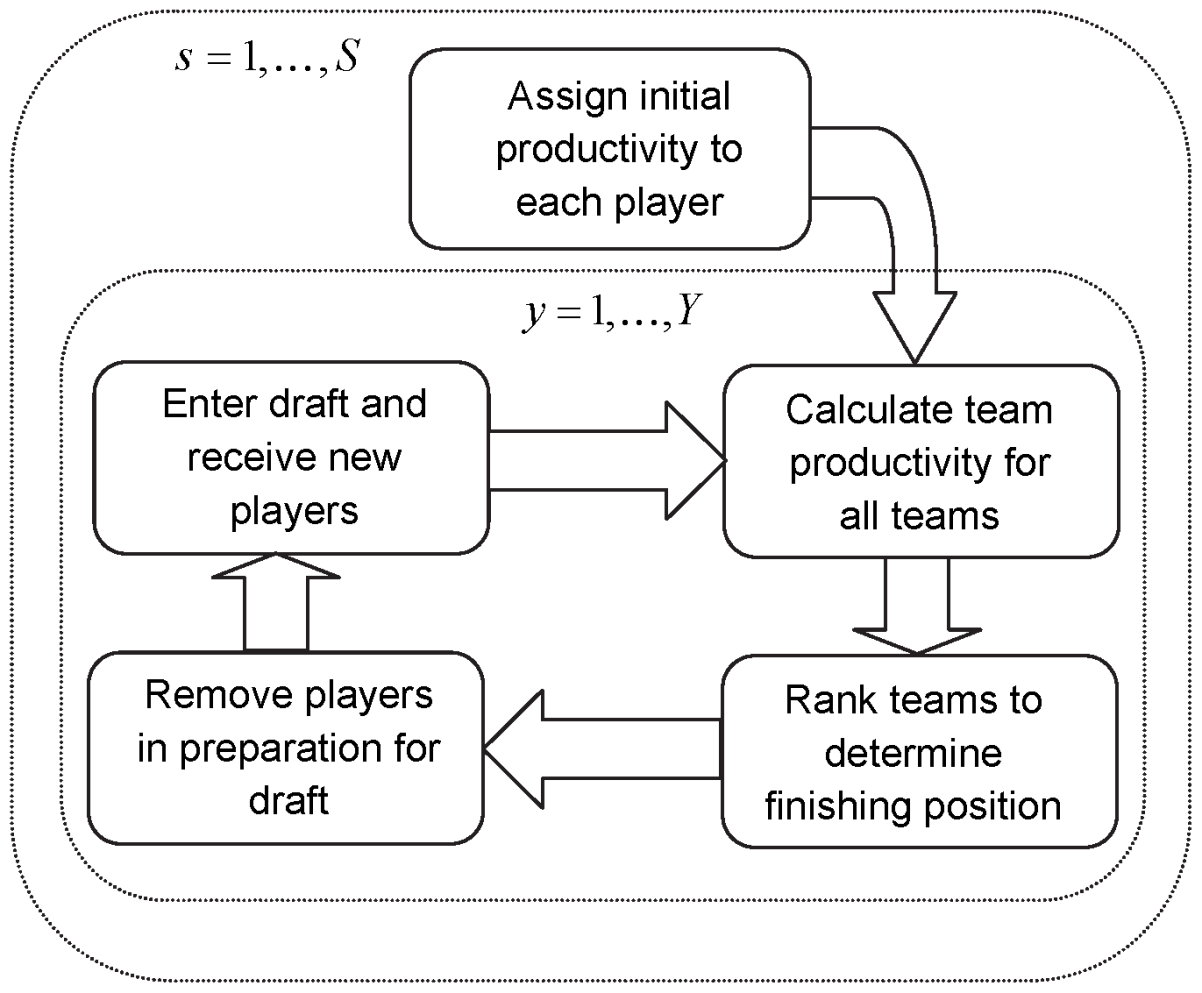
A diagrammatic representation of the major components of the player draft simulation model, iterated over a total of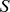 simulations and 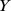 years.

The team productivity is, for a particular year, a function of the highest ranked player productivities over the number of players that are used on the field or court, and reflects a team’s strength. Here, the sum of player productivities is used to define the team productivity (as adopted by others [17], [19]). The club with the highest ranked team productivity is assumed to have won the premiership, and the team with the lowest team productivity is assumed to have finished last amongst all competing teams. Players are removed from team lists through retirement or de-listing, and new players are obtained through the particular draft system that the league has in place (for example, in reverse order to their finishing position or via a draft lottery) (Figure 1).

### Allocation of player productivity

Player productivity is a relative measure of a player’s ability over time and is assumed to be a function of the player’s draft pick and age. For a particular draft number, the productivity function is assumed to increase with age, reach a peak in performance and then decrease towards the end of the player’s career [30]–[32]. The productivity immediately prior to retirement age will not be zero as often players are still performing at a reasonable level, and decisions based around retirement can be due to mental and physical exhaustion or life-style choices, rather than ability. Estimates of peak performance vary between sports, with golfers generally peaking in their early thirties, baseball, NFL, and hockey players peaking in their late twenties, NBA players in their mid-twenties, and swimmers and tennis players in their early twenties [17], [30], [31], [33], [34].

Assuming clubs are attempting to choose the best players available at each draft pick, player productivity should decrease with draft number (see [7] for examples from the NBA and NFL). In reality, this may not always be true, as a club may decline a perceived better player in favour of one that suits the team’s current structure. The model also allows for choice error surrounding the productivity of a player, regardless of draft number. Clubs are assumed to be better at predicting high quality players, but due to choice error or unforseen and unpredictable events (such as long-term injury) high draft picks can have low productivity [16]. Similarly, it is less likely that a club will obtain a quality player with a low draft pick, but it is still possible. Clubs with a larger number of high order draft picks are assumed to have stronger playing lists; leading eventually to greater on-field success. Price et al. [16] show that clubs with the number one draft pick in the NBA have a higher than average winning percentage. Lenten [18] also suggests that the cycles of success seen in AFL teams are related to the reverse-order draft system providing better players to poor performing teams.

Potential datasets to define the productivity function include most valuable player awards, club best and fairest awards, numbers of games played, on-base percentage, number of assists, goals or points scored, or other statistically defined measures of player performance that can be tracked over a player’s career, such as ‘Dream Team’ or ‘Fantasy League’ points [30], [35], [36].

For the examples provided in this paper, we have chosen a truncated (at the maximum age) density function of the lognormal distribution to specify player productivity with age 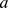, and a linear decline in productivity with draft number 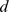. The maximum age is given by 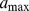, the age at which players retire, if they have not already been de-listed. The uncertainty associated with player productivity is assumed to follow a lognormal distribution, with uncertainty increasing with draft number. The lognormal density distribution was chosen for the productivity function as it has the requisite dome-shape and allows asymmetry about peak performance [33], [37]. Alternative functional forms could be considered [30], [32].

The player productivity function,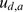, is given by
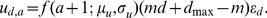
(1)


where
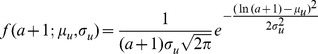



is the probability density function of the lognormal distribution, and defines the shape of the base productivity curve (when 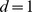 and 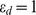) as a function of age 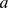 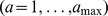



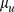
 is the shape parameter for the base productivity function


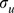
 is the scale parameter for the base productivity function


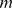
 is the slope of the linear relationship relating draft number to productivity, and is defined by
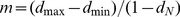
(2)


where 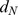
is the maximum draft number, and


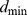
 is the minimum value in the linear relationship occurring when 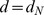



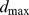
 is the maximum value in the linear relationship occurring when 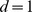



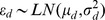
is a random variable from a lognormal distribution, with the mean of 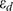 equal to 1. The location parameter 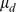 and scale parameter 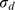 are defined by
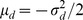
(3)

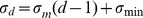
(4)

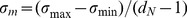
(5)


where 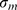 is the slope of the relationship defining the increased uncertainty with draft number,


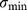
 is the scale parameter for the number 1 draft pick 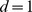



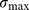
 is the scale parameter for the final draft pick 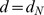


**Figure 2:**
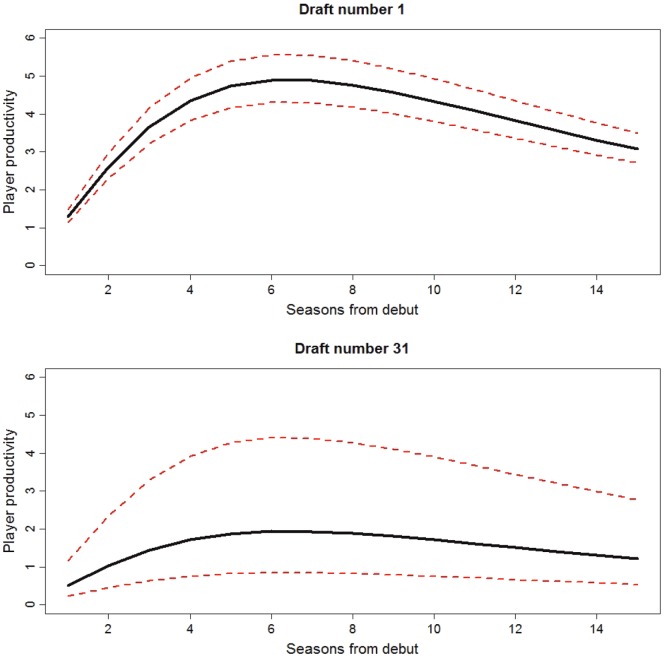
The player productivity as a function of seasons played (or age) for draft numbers 1 (top) and 31 (bottom) under the base-case parameter set. The median player productivity is shown in black, with 1^st^ and 99^th^ percentiles in red.

Figure 2 shows an example of the player productivity function as a function of age for draft picks 1 and 31 and illustrates how the median productivity is less for lower draft picks, and uncertainty regarding the productivity of a player increases with draft pick.

### The dynamic model

The model of Tuck and Whitten [25] assumes there are 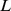 teams in a league, and that each team has 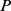 players on their team list (or squad) and an on-field team size of 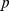. The initial ages for the player productivity functions for each team, 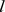, are chosen at random between ages 1 to 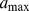 (ages are rescaled without loss of generality). The initial draft number for each player within a team is chosen at random between 1 and the maximum draft number 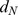, without replacement.

The team productivity, 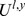, for team 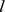 for a particular year 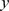 is the sum over the 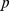 highest ranked player productivities of the team,



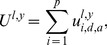
(6)


where 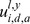 is the ranked player productivity for team 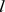, in year 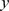, with draft number 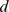 and age 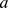, and the player productivity for player 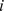 is greater than player 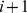,

The finishing positions are determined from the ranking of the team productivities. The premiership winner for the year is the team with the highest ranked team productivity. The order of allocation of draft picks is then determined by the particular player drafting system adopted by the league. A random lottery draft is considered so that comparisons can be more easily made between a reverse-order system that has a well defined-structure to player allocation, and a lottery draft that randomly allocates players to teams. With a random lottery draft (across all players and all rounds in the draft) each team is assigned draft picks randomly between numbers 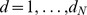. A reverse-order draft system has draft picks allocated in reverse order to the team finishing positions.

In order to make room for new players from the end-of-year draft, players are removed from team lists in one of two ways, (i) player retirements and (ii) de-listing. Each team must remove a minimum of 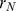 players from their playing list. As there are 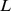 teams, this implies there must be at least 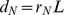 players in the draft. Firstly, players are removed that are of age greater than 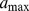 (player retirements). If there are greater than 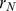 retirements, then all players recruited beyond the 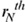 player receive the equivalent player productivity to the 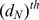 player drafted (with 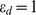). If there are less than 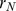 retirements in a team, then players with the lowest player productivity are removed (de-listed), until there are a total of at least 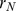 players marked for removal. These players are then removed from the playing list and the teams then enter the player draft.

New players are assigned to each team with their attributes defined by the player productivity function (equation 1) with (i) 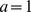, (ii) their particular draft number, 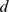 and (iii) uncertainty regarding player productivity defined by 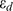. At the end of the draft process all teams have a full team list of 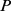 players once again. The model then moves to the next year,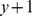, and the process of summing over ranked player productivities to determine the team productivity, premiership success and drafting begins again. A single simulation concludes in year 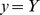 and each scenario is simulated 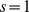 to 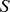 times.

### Under-performance

Once a team’s end-of-season team productivity has been calculated (equation 6), under-performance can be modelled by reducing the under-performing team’s productivity by the fraction 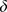, 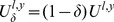
(7)


for all teams 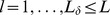 that have adopted an under-performing strategy. The parameter 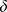 specifies the degree of tanking, that is, the larger the fraction, the more heavily a team tanks. Teams that have made the end-of-season finals or playoffs (from here on referred to as finals) have no incentive to under-perform, as might teams still vying for positions in the finals near the end of the season. Therefore the model assumes a team only under-performs if its ladder position is below 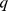. For example, in a 16 team league, if 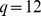 then a team adopting an under-performing strategy has its team productivity reduced by 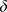 if it is ranked thirteenth or below. All teams from the league are then re-ranked, but with the adjusted team productivity of the tanking teams, and the premiership is decided according to the new team productivity rankings. An example where two teams adopt a tanking strategy is given in Table 1.

**Table 1:** An example of the re-ranking process where Teams 3 and 13 (bold italics) have adopted a tanking strategy for a particular simulation.

Team Number	Team Productivity	Rank	Adjusted Team Productivity	Adjusted Rank	Pick Number in the Draft
1	761	1	761	1	16
2	747	2	747	2	15
***3***	***746***	***3***	***746***	***3***	***14***
4	741	4	741	4	13
5	737	5	737	5	12
6	736	6	736	6	11
7	732	7	732	7	10
8	728	8	728	8	9
9	726	9	726	9	8
10	725	10	725	10	7
11	716	11	716	11	6
12	707	12	707	12	5
***13***	***705***	***13***	***635***	***15***	***2***
14	699	14	699	13	4
15	683	15	683	14	3
16	614	16	614	16	1

Team productivity gives a measure of a team’s strength, regardless of whether it tanks. Team 3 has made the finals and so does not tank in this year, whereas Team 13 under-performs by 10%, lowering its team productivity and lowering its ranking. Team 13 consequently has the potential to obtain a better player in the end-of-season reverse-order draft, as it now has pick number 2 instead of pick number 4 in the first round of the draft.

### Performance measures

Three performance measures of competitive balance that may be of interest to a league’s managing body are presented:

1. The proportion of premierships won by each team across years 41 to 140 and all simulations, 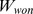

2. The mean over years 41 to 140 and all simulations 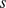 of the coefficient of variation (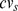) of the team productivities across all teams for a particular year,

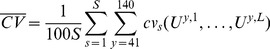
(8)

1. The mean over all simulations of the longest period for any team without premiership success, 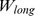 (over years 41 to 140).

The first measure allows a comparison of the success of teams across the league. In this way, the success or otherwise of a team adopting an under-performance strategy can be compared to other teams in the league. It is assumed that the larger this value, the greater perceived incentive there is to adopt an under-performing strategy. The first 40 years are removed from the summary statistic to eliminate any effects of the initial conditions. If a management objective is to ensure all teams in a league remain competitive, then the second measure of competitive balance provides a means of measuring the evenness of the competition, i.e. this value becomes smaller as the variation in team productivities becomes smaller. If a team tanks then the discounted team productivity is used (equation 7). This measure is similar to the Noll-Scully measure of competitive balance, where in our case team productivity is used as a proxy for winning percentage [6], [38]–[40]. This measure may be important to a league as the more even the competition, the more interest (and revenue) the game may generate as a spectacle [18]. The third performance measure gives the average longest duration for a team without a premiership over the simulation period, and provides an indication of how often teams become ‘stuck’ without premiership success.

The base-case parameter set is shown in Table 2, and is based upon an AFL-like league with 16 teams. The player productivity function and draft choice uncertainty are not estimated from data but are parameterised to capture the range of uncertainty that might be present (for example, we consider model sensitivity to no draft choice uncertainty (deterministic) and high levels of draft choice uncertainty).

**Table 2:** The base-case parameter set based upon an AFL-like competition.

Parameter	Description	Value
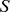	The total number of simulations	3000
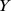	The total number of simulated years	140
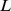	The number of teams in the league	16
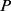	The number of players on the team list	40
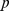	The team size	18
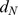	The number of players in the draft	80
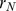	The minimum number of players per team replaced each year	5
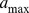	The maximum number of seasons per player (or re-scaled retirement age)	15
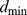	The deterministic value of productivity for draft number 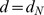	0.05
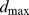	The deterministic value of productivity for draft number 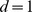	1.0
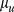	The shape parameter for the base productivity function	ln(14)
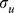	The scale parameter for the base productivity function	0.8
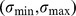	The scale parameters for 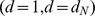	(0.05, 0.5)
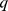	The finishing position below which a team adopts an under-performance strategy	12 (lower 4 teams)
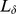	The number of teams adopting an under-performance strategy	1
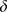	The discount to the team productivity for a team that under-performs	0.1

Alternative values used to explore model sensitivity are defined in the text.

## Results

**Figure 3:**
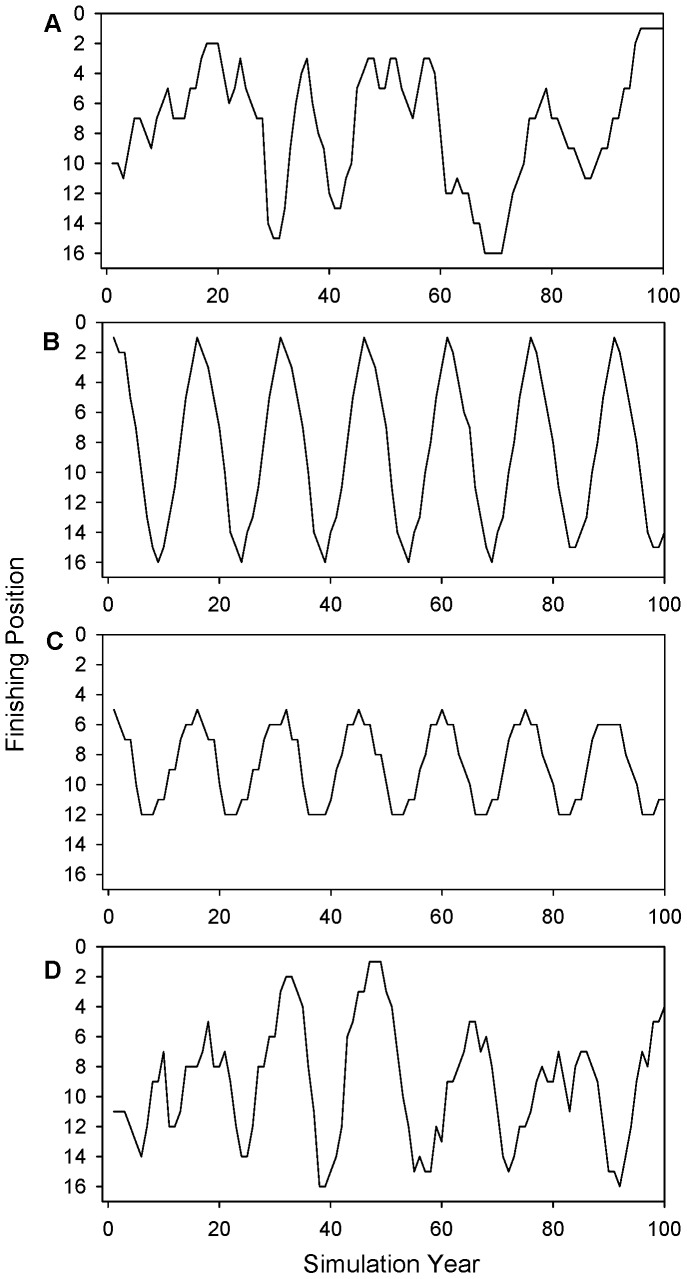
Finishing position of one of 16 teams in a league simulated over 100 years, for differing simulation scenarios: (a) random lottery draft system, (b) and (c) examples from a reverse-order draft system with no uncertainty regarding the ability of players drafted, 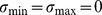, and (d) reverse-order draft system with draft choice uncertainty 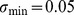 and 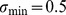.

Iteration of the dynamic model for 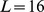 teams under the base-case parameter set and no error in the player productivity function, 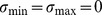, leads to an average proportion of premierships won of 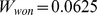 (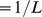) for both the random lottery and the reverse-order draft systems. Consideration of an individual simulation shows the random nature of team success with the lottery draft system (Figure 3(a)). However, a reverse-order draft system shows distinct cycles of team success (Figure 3(b)). Teams move from the bottom of the standings or premiership ladder, where they obtain better players in the draft, to the top, where they receive poorer players. However, this is contingent upon the initial conditions defined for each team. As has been conjectured, teams can become stuck in the middle of the ladder (Figure 3(c)), unable to obtain the good players necessary to win premierships, or poor players that may force it down the rankings so it can then obtain better players and join the premiership cycle. These teams do not bottom-out, but instead become stuck as middle ranked teams, often throughout the simulation period. The mean longest duration without premiership success is 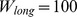
years, i.e. every simulation had at least one team that did not win a premiership over the simulation period.

Simulations with 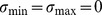 assume that teams receive players with known and deterministic productivity; the ability of a player is pre-determined once their draft number is known. If error is included in the productivity function, or more specifically, if the quality of a player received is not certain, then the deterministic cycles of premiership success and failure seen in Figure 3(b), become less apparent (Figure 3(d)). Over the long term, the proportion of premierships won remains at 0.0625, and while cycles are still evident, the frequency of success is less certain. Teams can go for long periods without premiership success (some over 100 years), however, due to the random nature of draft choices, teams in the middle of the ladder have a chance of obtaining better (or worse) than average players; allowing them to either rise up or drop down the premiership ladder and join the premiership cycle. This is reflected in the mean longest duration without a premiership, which reduces to 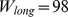 years (with 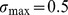 **)**. This remains a remarkably long time, which implies that (under the parameterisation of this model) some teams will naturally experience long periods of premiership drought, well beyond one in 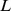 years.

### Under-performance

**Figure 4:**
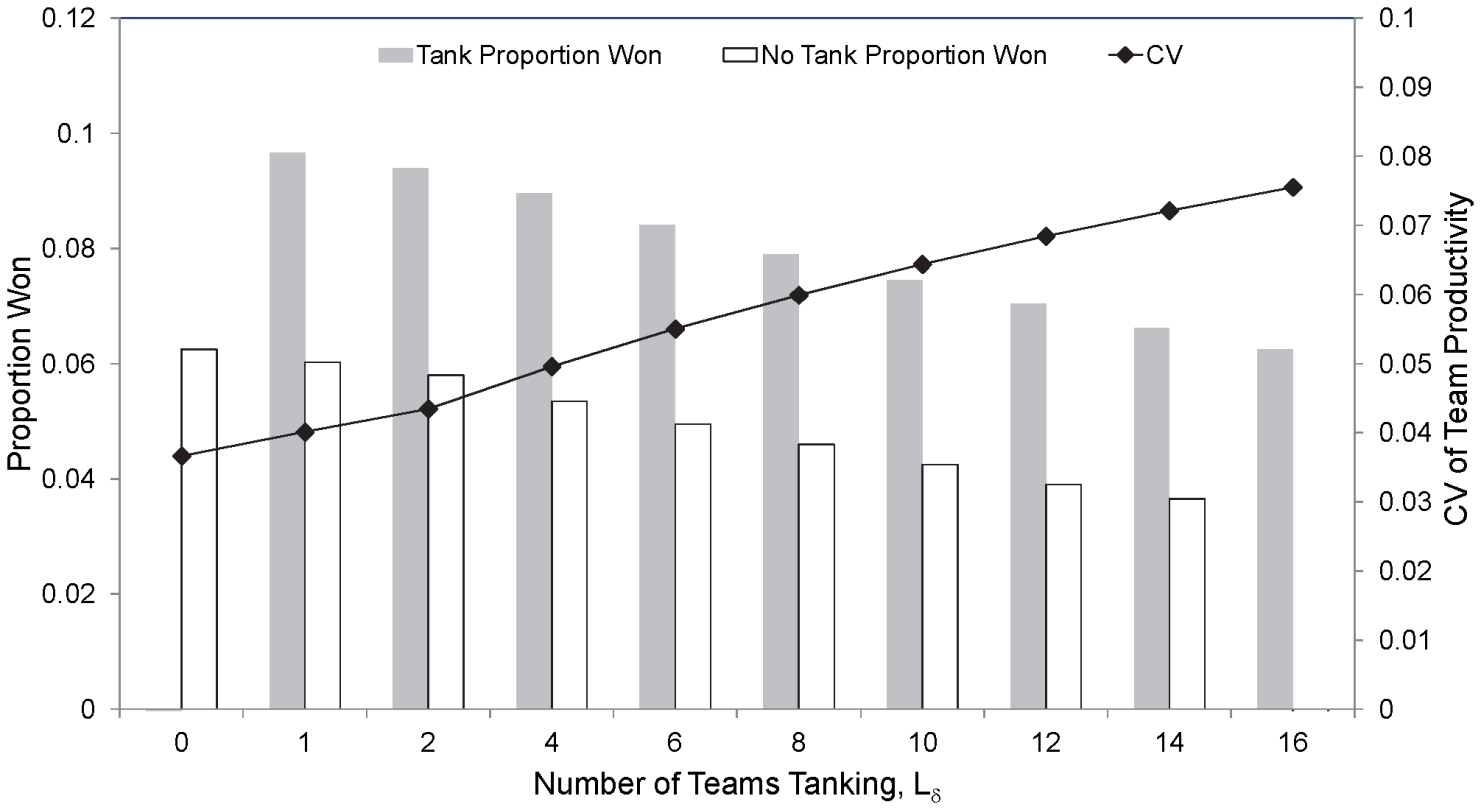
The proportion of premierships won for teams that tank (grey) and do not tank (white) as a function of the number of teams tanking. Also shown is the mean coefficient of variation of the team productivities for a particular year across all teams, years and simulations. This assumes the base-case parameter set.

With a random lottery based drafting system, a team that deliberately under-performs if it does not expect to make the finals does not benefit at all, namely the proportion of premierships won remains at 0.0625. However, with a reverse-order draft system a substantial increase in the proportion of premierships won can be obtained (Figure 4). The text that follows relates to a reverse-order draft system unless stated otherwise.

As the number of teams adopting the same tanking strategy increases (with 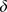 the same for each tanking team), the gain in premiership success decreases (as the spoils of success must be shared). Eventually there is no advantage whatsoever if all teams are tanking (Figure 4). Clearly, if teams in the league are tanking, then the share of premierships to teams that are not tanking reduces. As a team increases the amount by which it tanks (say the number of games it deliberately loses) that team’s gain in premiership success increases until such time as it cannot gain any more premierships by performing any worse (as it will be the lowest ranked team no matter how poorly performed it is during the season). For example, with 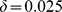 the proportion of premierships won is 0.09 compared to 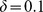 and 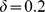 which are effectively the same at 0.1. The evenness of the competition reduces, as indicated by an increase in the mean cv of the team productivities, as the number of teams tanking increases (Figure 4) and the more they deliberately reduce their team productivity in order to drop down the premiership ladder. While teams that are likely to participate in finals are unlikely to tank, the model suggests that teams that only tank if lower down the premiership ladder will have a lower proportion of premierships than a team that is willing to tank if higher up the ladder (but not in the finals). For example, the proportion of premierships won if the tanking strategy adopted by a tanking team means that it only tanks if its ranked productivity (and therefore its non-tanking finishing position) in a particular year is in the bottom 2 (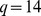) or bottom 8 (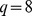) are 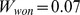
and 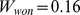 respectively.

**Figure 5:**
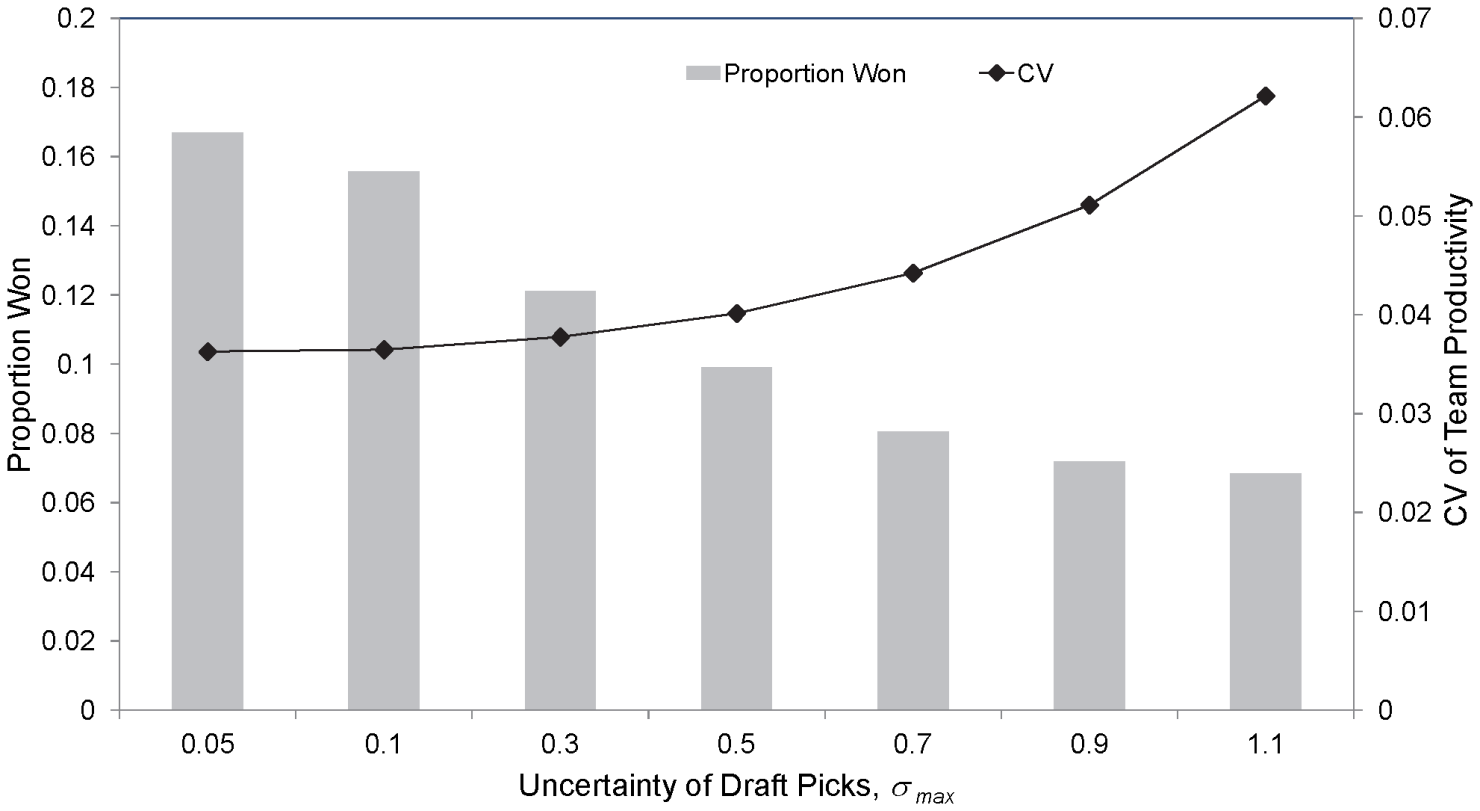
The proportion of premierships won for a single tanking team as a function of the degree of uncertainty regarding draft picks, 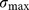. The mean coefficient of variation of the team productivities for a particular year across all teams, years and simulations is also shown. This assumes the base-case parameter set, with a single team tanking.

As the uncertainty regarding the ability of a player received in the draft increases (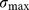
increases) the gain in premiership success of a tanking team decreases (Figure 5, [25]). If a team knows that it will acquire a marquee player with its top draft pick, then there is a strong incentive to use the system to obtain that player. However, if there is uncertainty regarding the ability of players in the draft, then this incentive diminishes. If the shape of the underlying player productivity curve moves from a younger-aged peak in performance of approximately 5.5 years from debut,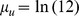, to an older-aged peak of approximately 12 years from debut, 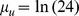, the proportion of premierships won by a single tanking team increases marginally (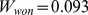 to 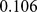), and the evenness of the competition decreases (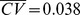 to 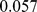).

As the number of teams,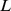, in the league increases, if no teams are tanking, then the proportion of premierships won decreases with 1/L. However, the relative gain in premierships increases for a tanking team. For example, under the base case parameterisation (with 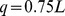 and 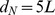), the relative increase in premierships won for a single tanking team when there are 12, 16, or 20 teams increases by a factor of 1.4, 1.6 and 1.7 respectively. This implies that the more teams there are in the league, the greater the incentive to tank.

**Figure 6:**
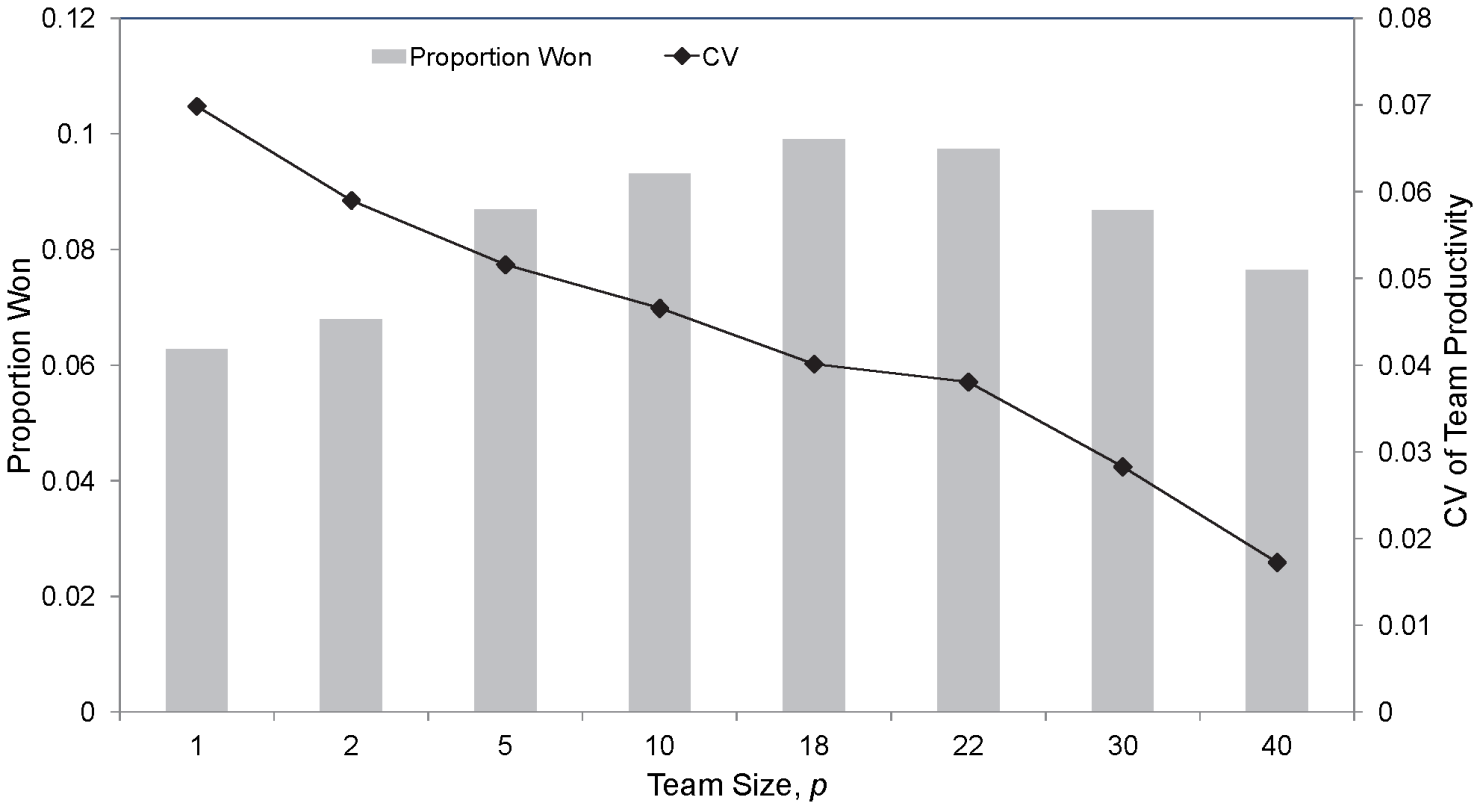
The proportion of premierships won for a single tanking team as a function of the number of players that constitute a team. The mean coefficient of variation of the team productivities for a particular year across all teams, years and simulations is also shown. This assumes the base-case parameter set, with a single team tanking.

In the deterministic case, the lower the team size, the greater is the incentive to obtain good players through tanking. This is because the influence of a marquee player becomes greater with fewer players on the field [12], [15]. However, this does not hold if uncertainty in draft picks is considered (with all else being equal). Figure 6 shows the proportion of premierships won as a function of team size, 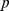, and where 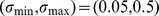. This figure illustrates that as the team size reduces to low levels, the incentive to tank reduces. To explain this result, if the team size is very small then it only takes one of the other non-tanking teams in the league to obtain, by chance, a better player or players for it to not have the best team productivity, and thus undo the perceived benefits of tanking. As the team size increases, the probability that the tanking team obtains a combination of players that are better than the other teams in the league increases (Figure 6).

## Discussion

This paper explores the dynamic characteristics of a win-maximising sports league through the stochastic simulation model of Tuck and Whitten [25]: ‘Sports Synthesis’. Sports Synthesis incorporates many of the key components of major sporting leagues, including player draft systems, player productivity, and draft choice error [12], [16], [19], [30], [33]. The non-equilibrium nature of the model allows an exploration of the dynamics of team success under different league characteristics [25], [29]. This papers shows, through sensitivity tests, that competitive balance can be influenced by variations to key league parameters (such as the uncertainty in identifying player ability, the number of teams in the league, the number of players in a team and deliberate under-performance of teams).

Simulations of a random lottery draft system and reverse-order draft systems show the random and cyclic nature of team success and failure. Sports Synthesis shows that long-term success rates are equally shared amongst all teams in competitions with reverse-order draft systems, but also shows that long periods of premiership drought for some teams can be an emergent property of these same systems.

In simulations where the ability of drafted players is known with certainty (zero draft choice error), Sports Synthesis demonstrates that teams can become fixed in the middle of league rankings for long periods. However, as uncertainty regarding the ability of players in the draft increases, this effect reduces, as a mid-ranked team may, by chance alone, select a high quality or poor quality player that will, one way or another, move their ranking away from mid-table.

A reverse-order draft system can lead to incentives to deliberately under-perform, as teams ‘lose to win’ by finishing near to or at the bottom of league tables in order to obtain higher quality players [12], [15]. The model presented here permits an exploration of the properties of a reverse-order draft system subject to deliberate under-performance strategies by one or multiple teams. We have shown that tanking can lead to a gain in on-field success, but this gain reduces (i) as more teams adopt a tanking strategy (ii) as the uncertainty regarding players’ ability increases (iii) with team size and (iv) as the number of teams in the league reduces.

These final points are worth further discussion. Borland, Chicu, and Macdonald [15] suggest that the NBA is more likely to have experienced tanking than the AFL because there is greater certainty around draft picks in the NBA and fewer players in a team [12]. The model presented here supports the first part of this conjecture but not necessarily the second. In the deterministic case, Sports Synthesis shows that tanking can provide high quality players that will strongly influence future team success. However, uncertainty surrounding the drafting process can almost completely remove this incentive: with small team sizes, there is an increased likelihood that other non-tanking teams will obtain a better player (or players) and, with so few players from which to choose, the tanking team’s productivity may not be sufficient to win the premiership. Sports Synthesis shows that the incentive to tank increases as the number of teams in a league increases (all else being equal). As such, while the number of players are less in the NBA, it may be the increased number of teams and the greater ability to pick quality players in the NBA that has driven incentives to tank in that competition.

There are clearly limitations to the Tuck and Whitten [15] model that could be improved upon. For example, a reverse-order draft system can be ineffective for competitive balance if players are able to move between clubs without, or with limited, restraint [4], [7]. The current model does not explicitly include player trading. In essence, the model implicitly assumes that a trade realises no gain or loss, in terms of team productivity, to either of the trading teams. However, in a modelling sense, tanking can be seen as an example of an inequitable trade whereby the tanking team obtains a better player than it delivers to the team that it replaced in the premiership ladder (as a consequence of tanking). Not surprisingly, the greater the inequity in the trade, the greater the competitive imbalance in the competition.

A key aspect of the Sports Synthesis model is the player productivity function (Figure 2). Several authors have statistically determined player value over time (productivity or aging curves) [30], [32], [41], [42]. While no data have been presented to estimate the player productivity function used in our paper, it is reasonable to expect that it is dome-shaped; increasing as players acclimatise and mature, reaching a peak in performance, and then tapering as players approach retirement. Assuming that club list managers are able to judge player talent to some degree, the model assumes that player productivity will decrease with increasing draft number, and that the ability of a club to pick quality players decreases with draft number (Figure 2). The form of the productivity function is somewhat subjective and can be chosen by the analyst in applying this model in a practical situation. However, we believe the general conclusions presented in this paper are likely to hold for a variety of alternative productivity functions. Further modelling work should consider an application to data from the AFL and other major sporting leagues. In this way, quantitative, rather than qualitative, measures of impacts on competitive balance can be considered and directly used to assist management decisions (regarding, for example, the particular draft system to employ [25]).

## Conclusion

This paper further explores the Tuck and Whitten [25] simulation model of a win-maximising sporting league, which is based upon a parameterisation of the Australian Football League. Non-equilibrium simulation models permit the exploration of stochastic dynamics in complex multi-parameter systems. Using a model of this type, Sports Synthesis, we were able to explicitly show the cyclic behaviour of team rankings under a reverse-order draft system and furthermore, show that teams can be caught in mid-ranked positions, with limited premiership success over very long periods. In addition, we have shown that a deliberate under-performance strategy (tanking) can substantially increase the frequency of premierships won in sporting leagues with reverse-order draft systems. Unfortunately, tanking also increases the disparity in ability (team productivity) between the weakest and strongest teams, and thereby reduces competitive balance. This has the potential to increase the number of games in the year that are uncompetitive; a result which may be harmful in terms of spectator interest and revenue to the league [18] (for counter arguments see [43], [44]).

Sporting managers face many complex challenges; Sports Synthesis, and models of its type, provide a mechanism to explore the impacts of management decisions prior to their implementation. These models can allow quantifiable comparisons of alternative draft systems, with respect to measures of competitive balance and the potential for undesirable team behaviours, and may help determine appropriate draft-related compensation for poor performing teams or new franchises.
